# Editorial Notes

**Published:** 1925

**Authors:** 


### Editorial Notes.

**The Royal Visit.**

THEIR Majesties the King and Queen are conferring a great honour on the University of Bristol as well as the city which it adorns when on June 9th His Majesty The King opens the new buildings, the magnificent gift of Sir George Wills, Bart., and of his brother the late Mr. Harry Wills, in memory of their father, Mr. H. Overton Wills, who did so much towards establishing the young University in its earlier years by his active co-operation with other illustrious citizens whose memory we cherish with gratitude. Deep regret is felt in that Mr. H. H. Wills cannot share with his elder brother, Sir George, the great joy of seeing their completed work formally inaugurated and honoured by the gracious presence of the King and Queen.

We have felt that such an occasion, a great day in the annals of the City of Bristol, calls for a special number of the Society's *Journal.*

**The Medical Faculty of the University of Bristol.**

THE complete equipment of the School of Medicine in Bristol, at least as regards its buildings, is an event that we cannot doubt foreshadows an expansion of scientific achievement and development worthy of the historic Bristol School in the past. Dr. J. A. Nixon, Professor of Medicine in the University, has but lightly touched on a few of the mile-stones carrying us back through some centuries to the time when to-day's achievement was but a prophetic conception, but within the limitations imposed has presented in outline the ample equipment the University affords for the education of the prospective graduate in the profession of medicine, and further for postgraduate research, and this we are convinced will appeal to all who are interested in medical education and progress.

We need make no apology by citing some words of John Ruskin, quoted by "Flambeau" in the *Wills's Works Magazine*: "Education is the leading of human souls to what is best, and making what is best of them. The training which makes men happiest in themselves also makes them most serviceable to others."

**Notes on the New Buildings of the University.**

By the courtesy of the Editor of the *Wills's Works Magazine* we are enabled to cite from Mr. G. H. Oatley's description of the new main buildings the following notes:—

"The building contains about fifty rooms, in addition to the Great Hall, Libraries and rooms for assembly.

"The original design was prepared at the close of 1912.

"The earlier buildings are Gothic, the flexibility of which style lends itself to obtaining windows of any size and shape required without the limitations imposed by symmetrical treatment.

"Furthermore, the necessity of obtaining on the site the maximum accommodation possible dictated the building of many floors.

"Again, the freedom of the Gothic style is more suited to a crowded site adjoining narrow thoroughfares.

"The Perpendicular phase of Gothic which was adopted is thoroughly English and suited to the climate and the temperament of the people. On these grounds the indigenous was followed rather than the exotic.

"The Tower is 57 ft. 6 in. square at the lower stage, with a height of about 215 ft. from the street, and is finished with an octagonal Belfry. As a dominating landmark of the neighbourhood its function is to compel attention to the existence of the University and, further to this end, it was designed to close the vista from the bottom of Park Street, the main artery between Bristol and Clifton.

"The Main Entrance is from Queen's Road and is in the base of the Tower. This leads into the Entrance Hall, 85 ft. by 32 ft., vaulted in fan vaulting, which rises to a height of 72 ft. from the floor. From this hall two parallel flights of stairs lead directly to the Great Hall, which is approached through a vestibule, or crush space, about 78 ft. by 20 ft., also vaulted in fan vaulting.

"The Great Hall—the body of which consists of six bays and measures 100 ft. by 50 ft., with a recess at the southern end containing two superimposed galleries and, at the north end, an apsidal recess with raised seats for Senate (or small orchestra) with organ behind it—is covered by a hammer-beam roof in English oak, ceiled at the collar, at a height of 55 ft.

"West of the vestibule to the Great Hall is a corridor leading to the Art Gallery, with which there are communicating doors for throwing the two buildings together on special occasions. Off this corridor, on the south side, is the Reception Room, 64 ft. by 33 ft. and 20 ft. high to the crown of the ornamental plaster ceiling. Its walls are panelled in oak; it has a Minstrel Gallery at one end and an Oriel Window at the other. Adjoining it is a Robing Room.

"The Council Room is duodecagonal, having a diameter of 41 ft., and is vaulted in stone. The room is panelled in oak with linen-fold panelling to the height of the window cills.

"The Library, 101 ft. by 34 ft. and 31 ft. high to the crown of the ceiling, which is of ornamental plaster, with pendants, has bookstacks arranged to form alcoves, and a gallery around it similarly planned.

" The Medical Library at ground floor level is of the same size as the Library last mentioned. In the Basement underneath this are the **rooms of the Medico-Chirurgical Society.**

" The Great Bell, cast by Messrs. John Taylor and Company, of Loughborough, weighs nearly ten tons. Its note is the lower E flat, and it is pronounced by experts in campanology to be, from the musical point of view, the finest E flat bell in Europe and, in all probability, the finest bell of its note ever cast ; the casting itself is perfect and the tone is resonant, pure, and of a fine rich musical quality ; the bottom octave of the chord (or hum note) is wonderful, giving forth a most impressive and powerful " boom," yet, withal, so round and musical that it is possible to stand close by and hear it with pleasure."

**Colston Research Society.**

THE Annual Dinner was held on Friday, May 22nd, at the University (Victoria Rooms), under the presidency of the Vice-Chancellor. The principal guests were the Right Hon. Lord Eustace Percy, Minister of Education, and Sir Ernest Rutherford, O.M., LL.D., F.R.S. There was an attendance of close on 100. The subscription list amounted to over £550, which was slightly in excess of the previous.

The Society continues to increase the number of its supporters and thereby to extend the range of its activities

